# Poster Session II A337 EVALUATING THE QUALITY OF IBS-RELATED CONTENT ACROSS MAJOR SOCIAL MEDIA PLATFORMS: A CROSS-SECTIONAL STUDY

**DOI:** 10.1093/jcag/gwaf042.337

**Published:** 2026-02-13

**Authors:** S Watson, V Premjeyanth, C Parker

**Affiliations:** University of Toronto, Toronto, ON, Canada; University of Toronto, Toronto, ON, Canada; University Health Network, Toronto, ON, Canada

## Abstract

**Background:**

Irritable bowel syndrome (IBS) is the most common disorder of gut-brain interaction, affecting 10-15% of the global population. Social media is a common source of health information and support for individuals with IBS. However, limited research has examined the quality of this content. Available studies suggest that content is often low quality and misaligned with clinical guidelines. Currently, no studies have conducted a cross-platform analysis of IBS related social media content.

**Aims:**

To systematically evaluate the quality and thematic content of IBS-related information across three major social media platforms. We hypothesized that the quality of IBS-related health information would be mediocre.

**Methods:**

We conducted a cross-sectional study of the top 50 most-engaged IBS-related posts on TikTok, Instagram, and YouTube (total n = 150), retrieved on June 29, 2025. Posts were assessed using the PRinciples for Health-Related Information on Social Media (PRHISM) rubric, a 13-item framework assessing the quality of health information shared on social media for non-expert audiences. Each principle was rated on a five-point Likert scale (0-4) and posts were categorized as Poor (0-25%), Mediocre (26-50%), Good (51-75%), or Excellent (76-100%). Two reviewers independently scored all posts, with discrepancies resolved by a third reviewer. A qualitative thematic analysis of posts was performed using Braun and Clarke’s six-phase approach.

**Results:**

Across all platforms, the mean PRHISM score was 41.32%, corresponding to a “Mediocre” quality overall. YouTube had the highest mean score lying at the upper boundary of “Mediocre” range and approaching “Good”, followed by Instagram and TikTok (Table 1). The lowest-scoring PRHISM principles were attribution (providing references or source links), referrals and support (referring users to additional resources), and complementary information (encouraging engagement with healthcare professionals). Key themes included patient symptom narratives, dietary strategies, promotion of unverified treatments, skepticism toward conventional care.

**Conclusions:**

This study demonstrates that the top IBS posts on TikTok, Instagram, and YouTube offer overall mediocre-quality health information, with YouTube performing at the upper end of this range. While social media can facilitate sharing insights about IBS, these findings provide important takeaways on the information patients are exposed to online. This underscores that social media may not be a reliable source of information about IBS despite its high rate of usage among patients.

**Funding Agencies:**

None

**A337 Table 1:** Mean PRHISM score and classification by platform

Platform	Mean Score (%)	Classification
YouTube	50.97	Mediocre-Good
Instagram	39.86	Mediocre
TikTok	33.12	Mediocre

